# Theoretical and Experimental Study on the Surface Microstructures of Polyimide in Ultra-Precision Fly-Cutting

**DOI:** 10.3390/polym17081099

**Published:** 2025-04-18

**Authors:** Jie Liu, Sheng Wang, Qingliang Zhao

**Affiliations:** Center for Precision Engineering, School of Mechatronics Engineering, Harbin Institute of Technology, Harbin 150001, China; liujieyusu@163.com (J.L.); zhaoqingliang@hit.edu.cn (Q.Z.)

**Keywords:** polyimide, ultra-precision fly-cutting, surface microstructures, maximum cutting thickness model, cutting force

## Abstract

Polyimide (PI) with surface microstructures has broad application prospects in aerospace, integrated circuits, and optical engineering due to its excellent mechanical properties, high thermal stability, and chemical resistance. Ultra-precision fly-cutting (UPFC) is a promising advanced technique for machining PI microstructures. However, few studies on the UPFC of PI materials are reported. In this study, the machining principle of UPFC is analyzed, and a comparative study of different processing strategies is conducted. The experimental results demonstrate that the climb cutting strategy is more suitable for PI microstructure machining, which can significantly reduce burr formation and achieve lower surface roughness. The theoretical models describing tool motion and predicting maximum chip thickness in UPFC are established, and the predicted chip thickness is consistent with the experimental results. Moreover, the influence of process parameters on the surface morphology and dimensional accuracy of microstructures is assessed through a series of experiments. The results indicate that cutting depth and step-over are the dominant factors influencing dimensional accuracy and surface roughness. Furthermore, the cutting force during UPFC is extremely small, only in the range of millinewtons (mN). In addition, the cutting force in the feed direction exhibits a high sensitivity to variations in process parameters compared to other directional components. This study provides theoretical guidance for the establishment of a theoretical model and the selection of UPFC process parameters for fabricating PI microstructures.

## 1. Introduction

Ultra-precision fly-cutting (UPFC) is an intermittent cutting method that has been extensively used for machining surface microstructures with high shape accuracy and low surface roughness, owing to its advantages of a high cutting speed and low material removal [1,2,3,4]. These surface microstructures are applied in the field of high-precision optical components, heat dissipation devices, and hydrophobic surfaces [5,6,7,8].

Currently, the most widely applied surface microstructures fabricated by UPFC include V-shaped grooves, rectangular grooves, and pyramidal structures [9,10,11,12,13,14,15]. Wang et al. [11] utilized UPFC technology to machine secondary micro-groove arrays on primary asymmetric surfaces made of copper as the base material, forming a repeating rib microstructure. This repeating rib structure enhanced thermal performance and minimized pressure drop. Cheng et al. [12] applied UPFC technology to achieve the high-precision fabrication of hydrophobic micro-grooves and micro-pillars on the bare surface of ring olefin copolymers in a single step. The experimental results demonstrated that droplets exhibited excellent sliding behavior on the bare micro-oriented groove surfaces of the olefin copolymer, highlighting the significant potential of such surfaces. Wu et al. [13] proposed a novel method for fabricating micro-optical waveguide molds based on UPFC, and successfully produced a micro-polymer optical waveguide mold for molding processes. Wang et al. [14] employed a 120° V-shaped diamond tool for transverse cutting via UPFC to fabricate a micro-pyramid mold, which exhibited an excellent surface quality and a high shape accuracy. Optical tests on the micro-pyramids replicated on PMMA through precision injection molding revealed clear and well-defined images. In addition, Zhang et al. [15] developed a novel fly-cutting system and manufactured optical components with overlapping micro-lenses featuring a secondary pyramidal structure.

The UPFC machining technique can been classified into two cutting methods according to the tool positioning. When the tool is aligned parallel to the spindle axis, the method is referred to as end-face fly-cutting [16,17,18,19,20,21]. Another type is known as radial fly-cutting, in which the tool is mounted perpendicular to the spindle in the radial direction [22,23]. By contrast, the characteristics of radial UPFC enable its broader range of applications [24,25,26,27,28]. As a result, many researchers have conducted in-depth studies on the cutting mechanisms and theoretical models of radial UPFC. Wang et al. [29,30,31] conducted in-depth studies on the material removal mechanism of UPFC, investigating both theoretically and experimentally the effect of workpiece material on surface roughness in UPFC. They found that elastic recovery significantly influenced the surface finish. Aluminum bronze, due to its hardness and rapid elastic recovery, had the greatest impact on surface quality, while machining copper resulted in the lowest surface roughness. Cheng et al. [32,33] proposed a purely geometric model to optimize machining conditions (tool tip geometry, spindle speed, cutting depth, feedrate, swing distance, and step-over) and cutting strategies (horizontal and vertical cutting), and to predict the surface roughness in UPFC. Kong et al. [34] developed various surface roughness models to predict and optimize surface generation, while considering cutting mechanics and cutting strategies. Zhang et al. [35,36,37] developed a geometric model to analyze the effect of tool wear on surface generation. The model represented the relationship between the geometry of the worn tool and the surface topography, enabling the monitoring of diamond tool wear. Zhao et al. [38] proposed a mechanical cutting force model that considered the material hardness, elastic modulus, uncut chip area, tool geometry, and the average value of the shear angle. This model effectively predicted the cutting force in the diamond fly-cutting of microstructure surfaces. S. To et al. [39,40] developed an analytical model to predict the main cutting force and thrust force in V-groove UPFC. They discussed the influence of machining conditions on cutting forces.

A large number of researchers have conducted in-depth studies on the cutting mechanisms, surface roughness, and tool wear in UPFC, primarily focusing on metallic and brittle materials. However, there is relatively little research on polymers, and there are clear differences between polymers and other materials. The viscoelasticity, low elastic modulus, limited thermal conductivity, and high thermal expansion coefficient of polymers pose significant challenges in fabricating microstructures with a high shape accuracy on polymer surfaces [41,42,43,44,45]. Furthermore, in UPFC, the material buildup during entry and exit, burr formation, as well as microstructure deformation and distortion occur along the edges of the microstructures, further leading to poor surface quality [46,47,48]. This presents significant challenges for UPFC in machining high-quality microstructures on polymer surfaces.

In this study, the micro-groove structures on polyimide surfaces were fabricated using UPFC and the influence of process parameters on the structure accuracy was investigated by theoretical analysis and experiments. First, the fundamental machining mechanism of UPFC was analyzed. Subsequently, the effects of key process parameters including spindle speed, feedrate, and depth of cut on surface quality (e.g., roughness, burr formation) and dimensional accuracy were systematically evaluated. Furthermore, a theoretical model based on the maximum chip thickness was developed to predict process limitations, and its validity was confirmed through experimental verification. The findings provided both theoretical insights and practical guidelines for microstructure processing on polymers using the method of UPFC.

## 2. Theoretical Analysis

### 2.1. Machining Principles

**Figure 1:**
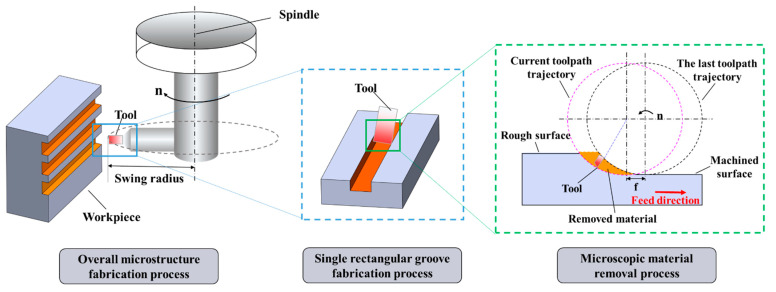
Schematic of the UPFC.

UPFC is an intermittent machining process in which a diamond tool rotates around a fly-cutting spindle. Typically, only one diamond tool is used, and the workpiece remains stationary. The tool intermittently enters and exits the workpiece surface through the movement of a linear axis. During the machining process, the tool’s linear velocity remains constant because the spindle speed and the tool’s swing radius (the distance from the tool tip to the fly-cutting spindle axis) are fixed [49,50,51]. Figure 1 illustrates a schematic of the UPFC.

### 2.2. Trajectory Method and Profiling Method

**Figure 2:**
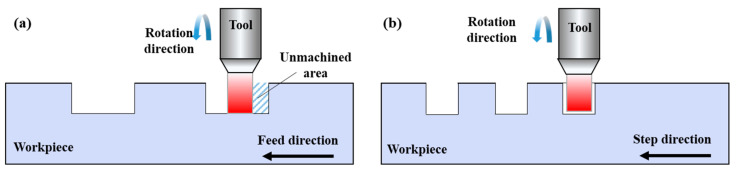
Fly-cutting machining methods: (**a**) trajectory method, (**b**) profiling method.

In UPFC, the formation of the workpiece microstructure morphology can be classified into two machining methods: the trajectory method and the profiling method, as shown in Figure 2. The trajectory method refers to the machining morphology on the workpiece being formed by the superposition and interference of the tool tip’s movement trajectory. In contrast, the profiling method refers to the workpiece morphology being entirely determined by the tool geometry, meaning that the surface morphology of the workpiece is replicated from the tool shape [52,53,54,55].

Both machining methods have their respective advantages and disadvantages. In terms of the tool, the trajectory method allows for the machining of microstructures of any size without requiring precise tool tip angles for the diamond tool. It achieves the desired surface morphology on the workpiece by superimposing multiple trajectories to achieve the required precision. In contrast, the profiling method demands high precision in tool preparation, as the tool must have high geometric accuracy to precisely match the required workpiece morphology. Only with such accuracy can the intended workpiece shape be successfully fabricated. Therefore, the geometric accuracy of the tool directly affects the machining precision of the workpiece. In terms of machining equipment, the trajectory method requires more complex machining paths, which impose higher requirements on the machine tools, typically necessitating multi-axis precision coordination; otherwise, machining errors may occur. On the other hand, the profiling method involves simpler machining paths, resulting in lower equipment requirements, provided that the machine tool’s positioning accuracy is maintained. In terms of machining efficiency, the trajectory method involves complex machining paths, which means that to machine the same microstructure on a workpiece, a longer machining time is required for the path superposition. In contrast, the profiling method only requires one pass to replicate the tool’s shape onto the workpiece. Thus, for the same microstructure and machining speed, the profiling method is significantly more efficient than the trajectory method. As a result, the profiling method is a more efficient machining approach. Considering these factors, the profiling method was ultimately chosen as the machining method for this study.

### 2.3. Cutting Strategy

#### 2.3.1. Horizontal and Vertical Cutting

**Figure 3:**
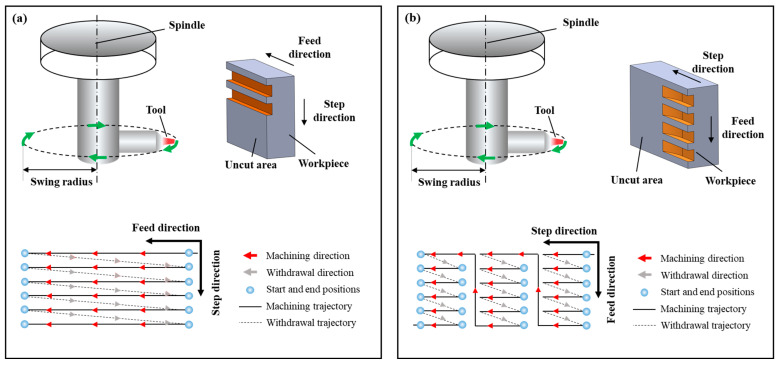
Machining strategy for UPFC: (**a**) horizontal cutting strategy, (**b**) vertical cutting strategy.

In UPFC, two cutting strategies exist: vertical cutting and horizontal cutting. During the cutting process, two main movements occur: feed motion and raster motion. Different suitable cutting strategies are required in UPFC, depending on the geometric features of the desired surface microstructure. Figure 3 shows the horizontal cutting strategy, where Figure 3a illustrates the relative position of the tool and workpiece under the horizontal cutting strategy and the tool’s machining trajectory. In the horizontal cutting strategy, the feed direction of the cutting tool is horizontal. After completing one cutting step, the diamond tool moves one step in the stepping direction. This process is repeated across the entire surface to achieve microstructure fabrication. Figure 3b illustrates the vertical cutting strategy, where the relative position of the tool and workpiece under the vertical cutting strategy and the tool’s machining trajectory are shown. In the vertical cutting strategy, the feed direction of the cutting tool is vertical, and the stepping direction is along the horizontal direction.

This study focuses on machining rectangular groove structures. When using the vertical cutting strategy, the tool must frequently engage and retract in the *Z* axis cutting depth direction, which reduces machining efficiency. In contrast, the horizontal cutting strategy allows for continuous machining along the horizontal direction, eliminating the time that would otherwise be spent on frequent tool engagement and retraction in vertical machining, significantly improving the machining efficiency. Therefore, considering the geometric characteristics of the microstructure being machined, the horizontal cutting strategy is the most suitable [32,56].

#### 2.3.2. Unidirectional and Bidirectional Cutting

**Figure 4:**
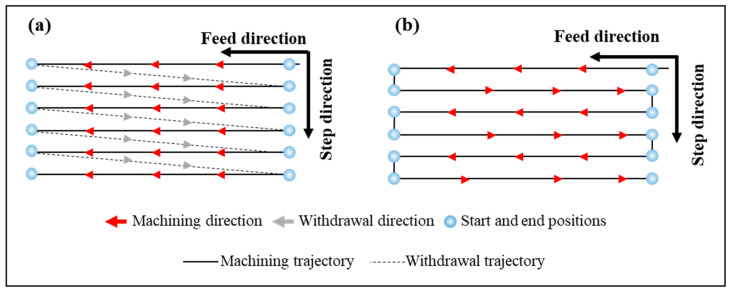
Different tool path planning in UPFC: (**a**) unidirectional, (**b**) bidirectional.

Based on the characteristics of the rectangular groove microstructure, the tool paths in UPFC can be classified into unidirectional cutting and bidirectional cutting, as shown in Figure 4. Figure 4a shows the tool machining along the feed direction until reaching the end of the workpiece. Then, the tool retracts and returns to the starting position during the idle travel, while the workpiece moves a step distance in the stepping direction for the next machining pass. This process is repeated until the entire surface of the workpiece is filled with rectangular microstructures, maintaining consistency in the tool trajectory. Figure 4b shows that at the end of machining one groove, the tool does not retract but instead moves a distance in the stepping direction. The tool then cuts the workpiece in the opposite direction of the previous groove machining, which results in a change in the tool trajectory between adjacent grooves, compromising the consistency of the structure [9]. Additionally, the fly-cutting axis used in this study is not integrated into the machine tool, which means that during bidirectional cutting, the tool’s rotational direction cannot be adjusted. As a result, the tool cuts the workpiece with its rear face, leading to poor machining results. Alternatively, the machining program needs to be stopped to adjust the rotation direction of the fly-cutting axis, which reduces efficiency and increases idle time. Therefore, a unidirectional cutting path is adopted in this experimental process.

#### 2.3.3. Climb and Conventional Cutting

**Figure 5:**
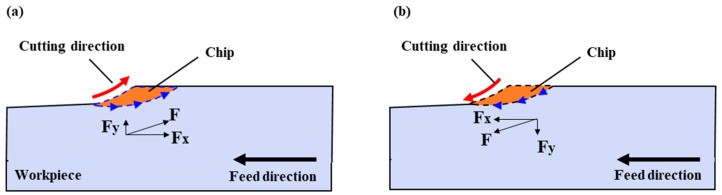
Material removal mechanism: (**a**) conventional cutting, (**b**) climb cutting.

In UPFC, two machining methods can be distinguished based on the relative motion direction between the tool and the workpiece and climb cutting and conventional cutting, as shown in Figure 5. In climb cutting, the cutting direction aligns with the feed direction, often resulting in improved surface quality. In conventional cutting, the cutting motion opposes the feed direction, potentially leading to higher cutting forces [57,58,59,60].

In climb cutting, the cutting thickness rapidly increases from zero to the maximum and then gradually decreases back to zero during each cycle. In conventional cutting, the cutting thickness gradually increases from zero to its maximum and then rapidly decreases back to zero. In conventional cutting, due to the small initial cutting depth, the tool’s cutting edge is not as sharp relative to the cutting depth. The presence of the tool’s edge radius prevents immediate cutting engagement with the workpiece. Instead, the tool slides and squeezes on the already machined surface, leading to significant surface hardening. This adversely affects the surface quality and accelerates tool wear [61,62]. Analyzing the cutting forces during the machining process, in climb cutting, the vertical component of the force (Fy) acts downward on the workpiece, which helps to secure the workpiece. In conventional cutting, the vertical component of the force (Fy) acts upward, creating a tendency to lift the workpiece. This is detrimental to securing the workpiece and may lead to machining vibrations, particularly for workpieces that are thin or have low stiffness. To verify the effects of climb and conventional cutting on microstructures, subsequent experiments were conducted.

### 2.4. Kinematic Analysis

Based on the above analysis of the UPFC principle, it can be concluded that the motion trajectory of the diamond fly tool is a circular rotational motion around the center of the fly tool spindle, while the workpiece simultaneously undergoes a linear feeding motion. Therefore, the motion trajectory equation [63] of the fly tool relative to the workpiece is:

(1) $$\left\{\begin{array}{l} y=R+R\cos(2\pi \mathrm{nt})\\ x=R\sin(2\pi \mathrm{nt})+v\cdot t \end{array}\right.$$

In the equation, R is the distance from the tool tip to the center of the fly tool spindle (152.69 mm), n is the spindle speed, v is the feedrate, and t is the fly tool operation time.

**Figure 6:**
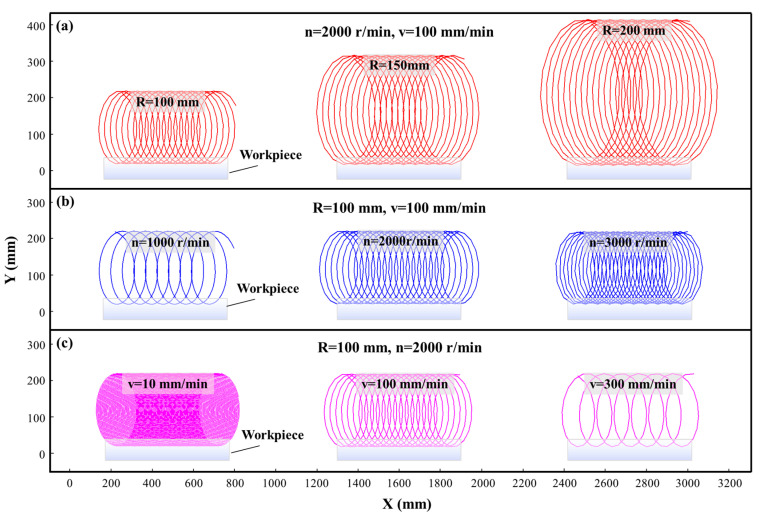
Cutting trajectories for different process parameters: (**a**) swing radius R, (**b**) spindle speed n, (**c**) feedrate v.

Based on the cutting trajectory (Equation (1)) of the fly tool relative to the workpiece, the cutting trajectories of UPFC under different parameters are calculated using Origin, as shown in Figure 6. From the figure, it can be observed that the cutting trajectory of the fly tool relative to the workpiece consists of a series of elliptical cycloids. Furthermore, three main machining parameters, R, n, and v, are found to influence the cutting trajectory of the fly tool in a systematic manner.

As shown in Figure 6a, when the radius R increases, the curvature radius of the cutting trajectory on the workpiece becomes larger, making the trajectory smoother. For the same cutting depth, the marks on the workpiece become longer. This implies that when the diamond tool is processing the workpiece, the cutting amount per unit time is lower compared to a smaller cutting radius. Therefore, increasing the cutting radius R increases the curvature radius of the cutting trajectory, reducing the cutting volume. However, a larger tool size makes it more challenging to maintain dynamic balance, potentially inducing vibrations that negatively impact both machining quality and system stability [64].

As shown in Figure 6b, when the spindle speed n increases, the cutting trajectories become denser over the same toolpath distance, meaning the toolpath is more closely spaced. For the same cutting volume, a higher spindle speed results in more tool passes, which obviously reduces the material removal rate per pass. Simultaneously, the cutting linear speed s increases, as s = 2πRn. However, an inevitable consequence of increasing the spindle speed is that it raises the imbalance in the machining system, leading to larger vibrations, which can damage the machine tool and increase machining errors [65,66]. As shown in Figure 6c, when the feedrate v decreases, the cutting trajectories become denser, similar to the effect of increasing the spindle speed. This leads to an increased number of tool passes within a given feed distance, reducing the cutting amount per pass. The key difference, however, is that the forward distance becomes shorter, which results in a decrease in the machining efficiency. By comparing Figure 6a–c, it can be concluded that, in UPFC, when considering machining efficiency, the feedrate should not be too small, as it is the only parameter that directly affects the machining efficiency.

### 2.5. Maximum Cutting Thickness Model

#### 2.5.1. Chip Thickness Model and Theoretical Calculation

**Figure 7:**
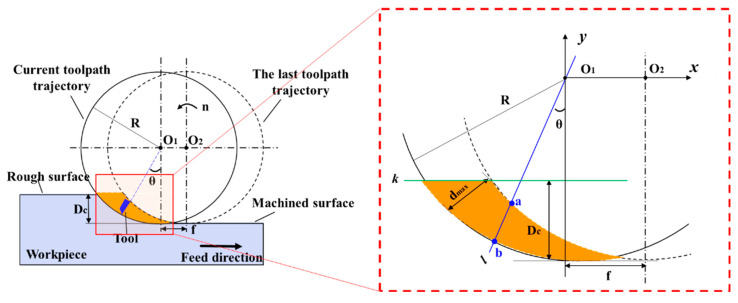
Schematic of the maximum chip thickness in UPFC.

Figure 7 illustrates the chip formation in UPFC. Upon observation, it can be seen that the chip is formed by the cutting actions of two successive tool passes, with the tool paths represented by circles O_1_ and O_2_. The orange region of the chip is defined by circles O_1_ and O_2_, along with the straight line k. The chip thickness is represented by the segment ab, which is obtained by the intersection of line l passing through the center O_1_ with the two circles. As the angle changes, the length of segment ab also varies. To better represent the relationship between the chip thickness and the angle, the center of circle O_1_ is set as the origin of the coordinate system, and the following calculations are performed:

The functional expression of the straight line l is given by:

(2) $$y=\frac{x}{\tan\theta }$$

The functional expression of the circle O_1_ is given by:

(3) $$x^{2}+y^{2}=R^{2}$$

where R represents the swing radius of the tool.

The mathematical expression for the circle O_2_ is:

(4) $${\left(x-f\right)}^{2}+y^{2}=R^{2}$$

where f represents the step-over, which is the distance of the feed direction change between two consecutive tool cuts.

The functional expression of line k is given by:

(5) $$y=R-D_{c}$$

where $D_{c}$ represents the cutting depth.

The length expression for the line segment ab is:

(6) $$d=\sqrt{{{(x}_{b}-x_{a})}^{2}{+(y}_{b}-y_{a}{)}^{2}}$$

The length d of the line segment ab is defined by the coordinates of points (x_a_, y_a_) and (x_b_, y_b_).

By substituting Equations (2)–(5) into Equation (6), the expression for the length d is obtained as follows:

(7) $$d=\sqrt{(f-\sqrt{R^{2}-(R-D_{c}{)}^{2}}+\mathrm{Rcos}\theta {)}^{2}+(R-D_{c}+\mathrm{Rsin}\theta {)}^{2}}$$

To find the value of θ corresponding to the maximum of d, we can differentiate the expression with respect to θ and find the points where the derivative equals zero. This is because when the derivative is zero, a local maximum or minimum may occur. To simplify the calculation, we will differentiate d^2^ (the square of d), as it has the same maximum as d. The definition is as follows:

(8) $$D(\theta )=(f\;-\sqrt{R^{2}-{\left(R-D_{c}\right)}^{2}}+\mathrm{Rcos}\theta {)}^{2}+(R-D_{c}+\mathrm{Rsin}\theta {)}^{2}$$

The derivative of θ is taken as follows:

(9) $$\frac{D(\theta )}{d(\theta )}=2(f\;-\sqrt{R^{2}-{\left(R-D_{c}\right)}^{2}}+\mathrm{Rcos}\theta )(-\mathrm{Rsin}\theta )+2(R-D_{c}+\mathrm{Rsin}\theta )(\mathrm{Rcos}\theta )$$

The derivative is set to be equal to zero:

(10) $$(f\;-\sqrt{R^{2}-{\left(R-D_{c}\right)}^{2}}+\mathrm{Rcos}\theta )(-\mathrm{Rsin}\theta )+(R-D_{c}+\mathrm{Rsin}\theta )(\mathrm{Rcos}\theta )=0$$

Solving this equation yields the angle θ that maximizes d.

(11) $$\theta =\arctan\frac{R-D_{c}}{f\;-\sqrt{R^{2}-{\left(R-D_{c}\right)}^{2}}}$$

Therefore, the value of the line segment ab is maximized, which corresponds to the maximum cutting thickness at this point.

#### 2.5.2. Influence of Process Parameters on the Maximum Cutting Thickness

By combining Equations (7) and (11), the expression for the maximum chip thickness can be obtained.

(12) $$d_{\max}=R-\sqrt{{\left(R-D_{c}\right)}^{2}+{\left(\sqrt{R^{2}-{\left(R-D_{c}\right)}^{2}}-f\right)}^{2}}$$

where $f=\frac{\nu }{n}$, v is the federate, n is the tool rotational speed, R is the tool rotation radius, and D_c_ is the cutting depth. The formula for further variation is given by:

(13) $$d_{\max}=R-\sqrt{{\left(R-D_{c}\right)}^{2}+{\left(\sqrt{R^{2}-{\left(R-D_{c}\right)}^{2}}-v/n\right)}^{2}}$$

According to Equation (13), the maximum cutting thickness of the chip is related to the tool’s rotational radius R, the tool rotational speed n, the feedrate v, and the cutting depth D_c_. To better understand the relationship between each variable and the maximum chip thickness, specific values for each parameter are selected and presented.

**Figure 8:**
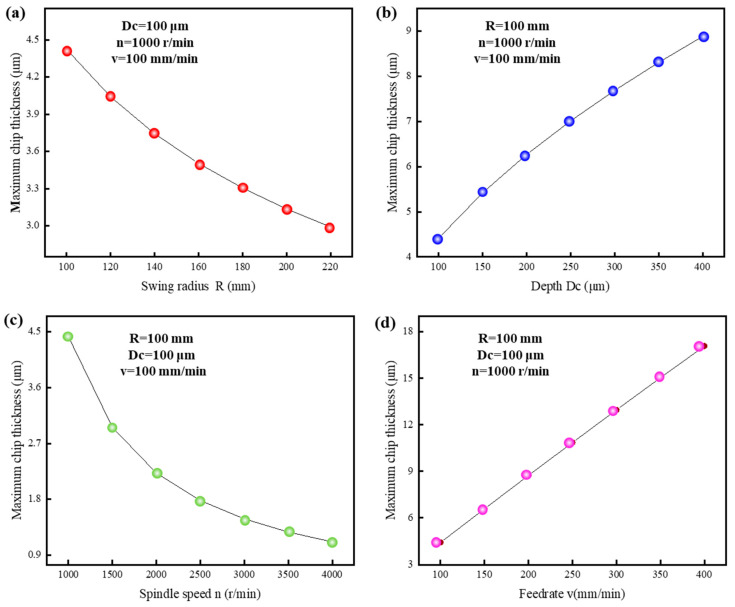
Influence of different process parameters on maximum chip thickness: (**a**) rotational radius, (**b**) cutting depth, (**c**) spindle speed, (**d**) feedrate.

Figure 8 shows the influence of different process parameters on the maximum chip thickness. In Figure 8a, it can be observed that as the tool rotational radius increases, the maximum chip thickness gradually decreases, with the rate of decrease becoming slower. At the same time, an excessively large tool rotational radius may increase the vibration and imbalance. Therefore, a rotational radius of approximately 150 mm is considered optimal. In Figure 8b, it can be observed that as the cutting depth increases, the maximum chip thickness gradually increases. When the cutting depth is less than 250 μm, the increase in maximum chip thickness is relatively rapid, while beyond 250 μm, the rate of increase in the maximum chip thickness slows down. From Figure 8c, it can be observed that as the spindle speed increases, the reduction in maximum chip thickness gradually decreases. In the low-speed range, increasing the spindle speed results in significant benefits, with the maximum chip thickness decreasing rapidly. When the spindle speed is between 1000 r/min and 3000 r/min, the maximum chip thickness ranges from 1.5 μm to 4.5 μm. When the spindle speed exceeds 3000 r/min, further increases in speed continue to reduce the cutting thickness. However, excessively high speeds can exacerbate dynamic imbalance and increase tool wear, negatively affecting machining quality. Moreover, the benefits of increasing speed further diminish. Therefore, in practical machining, a spindle speed range of 1000 r/min to 3000 r/min is recommended. Figure 8d shows that the feedrate has a linear effect on the maximum cutting thickness. For every 50 mm/min increase in the feedrate, the maximum cutting thickness increases by approximately 2 μm. Compared to the other three parameters, the feedrate has the greatest impact on the maximum chip thickness [67,68,69,70].

Based on the above analysis, the feedrate is linearly related to the maximum cutting thickness. To balance processing efficiency, a smaller feedrate should be selected whenever possible. When the cutting depth is small, the maximum cutting thickness is highly sensitive to changes in cutting depth. Additionally, increasing the rotational speed in the low-speed region offers significant benefits.

## 3. Materials and Methodology

### 3.1. Materials

**Figure 9:**
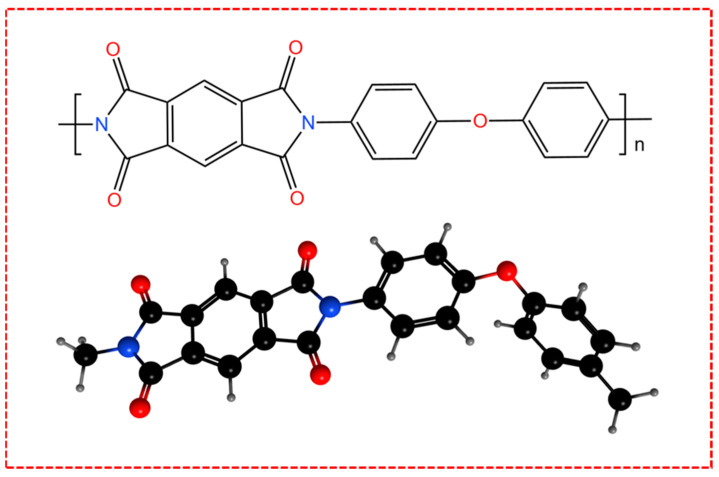
Schematic diagram of PI structure.

PI (Yusheng Hardware and Plastic CNC Precision Processing Game Company, Hong Kong, China), composed of aromatic and imide rings (Figure 9), exhibits superior thermal stability (>260 °C service temperature), mechanical properties (high modulus, low creep), and chemical/radiation resistance, as shown in Table 1. Its unique electron delocalization enables its excellent dielectric properties, facilitating its application in flexible electronics, aerospace, and microelectronics.

### 3.2. Experimental Setup

**Figure 10:**
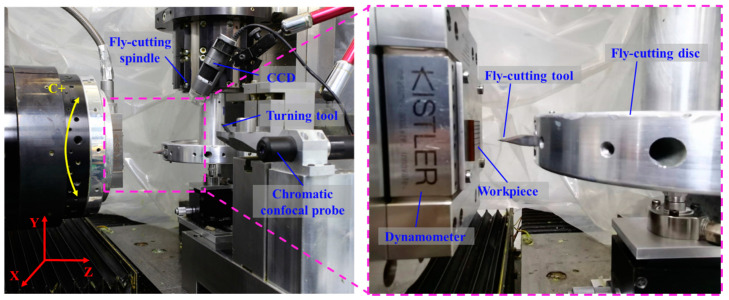
UPFC machining system.

The UPFC is used to machine rectangular groove structures with an equal depth on the surface of PI, where the groove width is 70 μm and the rib width is 100 μm. Figure 10 illustrates the ultra-precision machining system, which consists of three linear axes, an air-bearing spindle, and a liquid hydrostatic spindle. The workpiece is fixed on the air spindle using a fixture, and the diamond tool is connected to the high-speed rotating liquid hydrostatic spindle through the tool. The linear motion of the *x*, *y*, and *z* axes are employed to achieve the machining of the rectangular groove microstructures. Table 2 shows the machine parameters for the ultra-precision machining system, while Table 3 displays the processing parameters for UPFC in the machining of the rectangular groove microstructures. In order to avoid the randomness of the experimental results, each machining parameter is tested in triplicate. Considering the significant influence of temperature on the viscoelasticity of PI, cutting fluid is used during machining to dissipate excess heat and prevent temperature rise, thereby ensuring the machining accuracy of the microstructure.

### 3.3. Characterization and Evaluation

**Figure 11:**
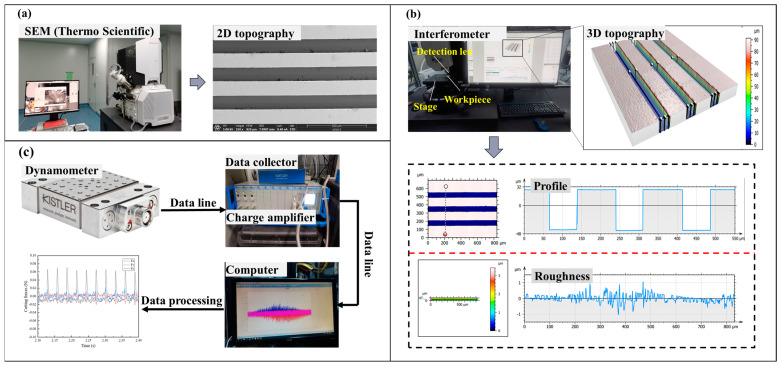
Testing equipment: (**a**) SEM, (**b**) Interferometer, (**c**) force sensor.

As shown in Figure 11, the morphology of the rectangular groove microstructure on PI was characterized using a scanning electron microscope (SEM, Thermo Scientific, Thermo Fisher Scientific Inc, Bragg, Czech Republic). The three-dimensional morphology, cross-sectional profile, and surface roughness of the rectangular groove microstructure were obtained using a white light Interferometer (Interferometer). The three-axis forces applied to the workpiece during the machining process were measured using a Kistler 9119AA2 force sensor (Kistler Group, Winterthur, Switzerland). Table 4 presents the basic performance parameters of the Interferometer.

## 4. Results and Discussions

### 4.1. Analysis of Climb Cutting and Conventional Cutting

**Figure 12:**
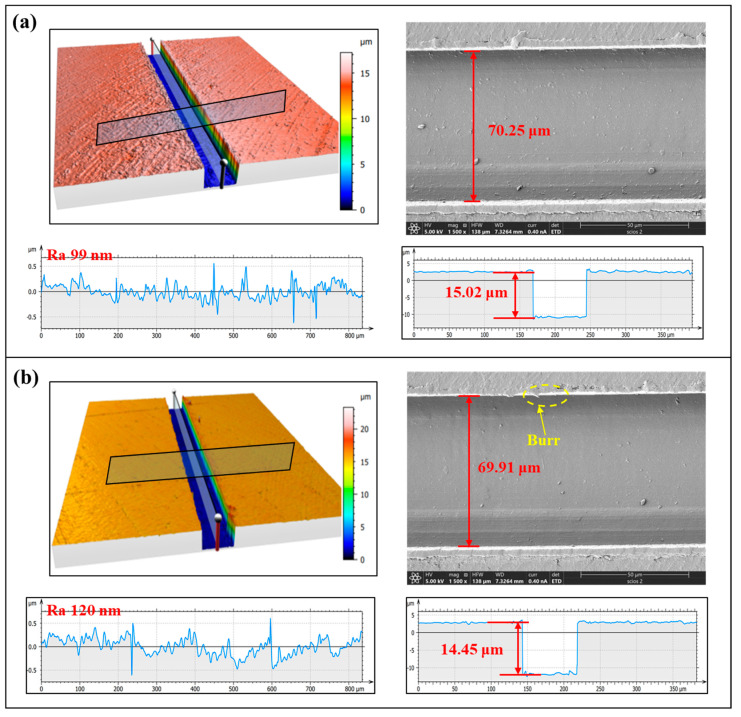
Surface morphology: (**a**) climb cutting, (**b**) conventional cutting.

Figure 12 shows the microstructural morphology of the rectangular grooves under climb cutting and conventional cutting conditions. The process parameters are a cutting depth of 15 μm, spindle speed of 2100 r/min, and feedrate of 30 mm/min. Analysis reveals that while conventional cutting yields better dimensional accuracy, climb cutting results in better burr formation and surface roughness. Due to the nature of the PI material, burr formation is more prone to occur during ultra-precision planing [41,71]. This is because during ultra-precision planing, the material is pushed by the tool and removed by an inclined upward force. In this process, the material experiences an upward tensile force (Fy), which causes it to elongate and neck. Once the material exceeds its fracture strength, it breaks, resulting in burr formation. The same principle applies to conventional cutting. In contrast, during climb cutting, the force applied by the tool to the material is directed downward, resulting in compressive force (Fy) on the material [72,73,74]. This reduces the likelihood of tensile forces causing burr formation. The force distribution in climb and conventional cutting is shown in Figure 5. Additionally, under the same processing conditions, the surface roughness in climb cutting is 21 nm lower than in ordinary cutting. This is because, during climb cutting, the downward component of the cutting force (Fy) compresses the bottom of the groove, effectively restraining the formation of surface defects and reducing the height of residual tool marks. As a result, the machined surface becomes smoother and flatter, exhibiting significantly reduced roughness. Moreover, the compressive effect of Fy may also enhance the material’s deformation behavior, promoting more uniform material flow and further contributing to surface quality improvement. These factors collectively lead to a finer surface finish in climb cutting compared to ordinary cutting [75].

In UPFC, the diamond tool rotates periodically, engaging and disengaging with the workpiece surface. During the rotary cutting process, the cutting force exhibits periodic fluctuations, with the periods being related to the spindle’s rotational speed, as given by the following equation:

(14) $$t=\frac{60}{n}$$

**Figure 13:**
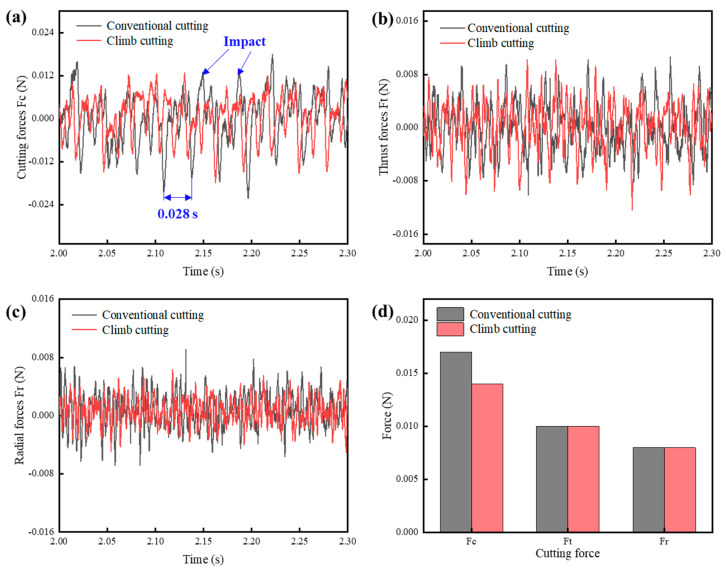
Cutting forces in climb and conventional cutting: (**a**) cutting forces, (**b**) thrust forces, (**c**) radial forces, (**d**) comparison of cutting forces.

In this equation, t represents the period of the rotary cutting process (s), and n is the spindle speed (r/min). Given that n = 2100 r/min, the period length of the rotary cutting process can be calculated as 0.028 s, which is consistent with the fluctuation period of the force shown in Figure 13. Each peak in the figure corresponds to the moment when the tool begins to engage the workpiece. By comparing the cutting forces in the two cutting modes, the thrust force and radial force are found to be nearly identical in both of the cutting directions. However, it is found that in terms of cutting force in the feed direction, the cutting force for conventional cutting is significantly greater than for climb cutting. This difference is due to two main factors. First, during conventional cutting, the rotation direction of the tool is the opposite to the motion direction of the workpiece. When the tool and workpiece make contact, the impact momentum is higher, making the tool more prone to breakage. In contrast, during climb cutting, the tool and workpiece move in the same direction, resulting in lower instantaneous impact forces. On the other hand, as introduced in Section 2.3.3, at the beginning of conventional cutting, the tool removes less material, and the tool’s cutting edge cannot be considered perfectly sharp. The tool does not immediately cut into the workpiece, but instead slides and presses against the already machined surface, resulting in higher friction forces on the tool. After comprehensive analysis, this study concludes that climb cutting is more suitable for microstructure machining, considering factors such as material properties, surface roughness, cutting forces, and tool wear angles. Therefore, in the subsequent research, climb cutting will be used as the machining method.

### 4.2. Microstructure Morphology and Profile

**Figure 14:**
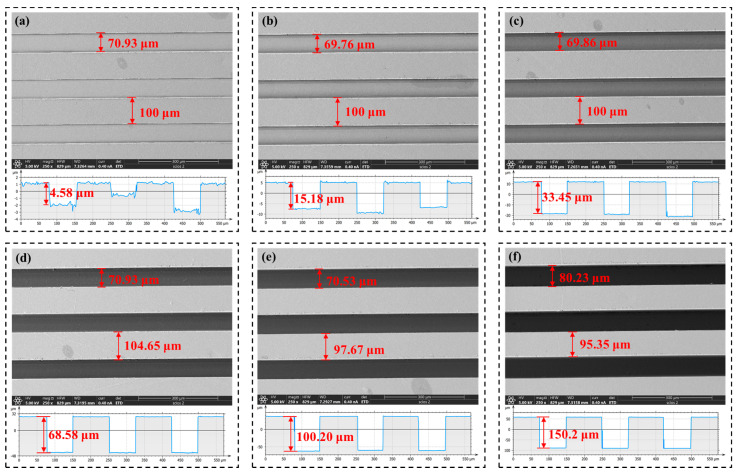
Microstructure morphology at different cutting depths: (**a**) 5 μm, (**b**) 15 μm, (**c**) 35 μm, (**d**) 70 μm, (**e**) 100 μm, (**f**) 150 μm.

**Figure 15:**
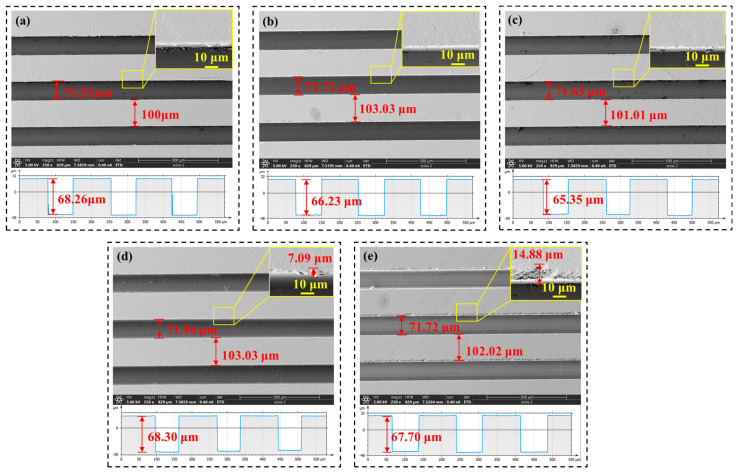
Microstructure morphology at different feedrates: (**a**) 10 mm/min, (**b**) 30 mm/min, (**c**) 60 mm/min, (**d**) 100 mm/min, (**e**) 300 mm/min.

**Figure 16:**
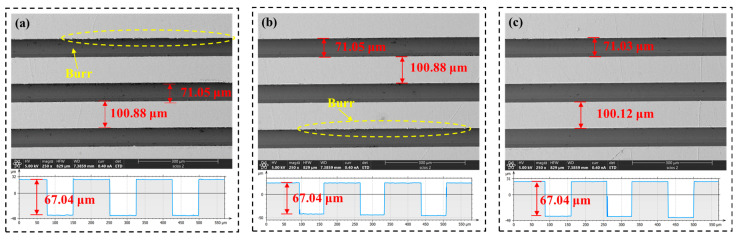
Microstructure morphology at different spindle speeds: (**a**) 2100 r/min, (**b**) 2600 r/min, (**c**) 3100 r/min.

Figure 14, Figure 15 and Figure 16 show the microstructure of the rectangular grooves under different process parameters. The SEM images and profile diagrams of the microstructure reveal that the rectangular grooves are clear, well formed, and are free from significant defects. In Figure 14d, it can be observed that when the cutting depth is below 70 μm, the groove width and rib width of the microstructure are in good agreement with the theoretical values. However, starting from a cutting depth of 70 μm, as the cutting depth increases, the rib width of the rectangular groove gradually decreases, while the groove width increases. At a depth of 150 μm, the actual groove width reaches 80.23 μm, which is 1.15 times the theoretical groove width (70 μm). This is due to the large cutting depth causing significant compressive forces on both sides of the groove, leading to the plastic deformation of the material along the groove edges. Below a cutting depth of 70 μm, it is observed that the actual groove depth of the rectangular grooves is slightly smaller than the theoretical depth, which is attributed to the material’s elastic recovery. From Figure 15, it can be seen that as the feedrate increases, defects appear along the groove edges. At feedrates of 100 mm/min and 300 mm/min, the defects at the groove edges are 7.09 μm and 14.88 μm, respectively.

**Figure 17:**
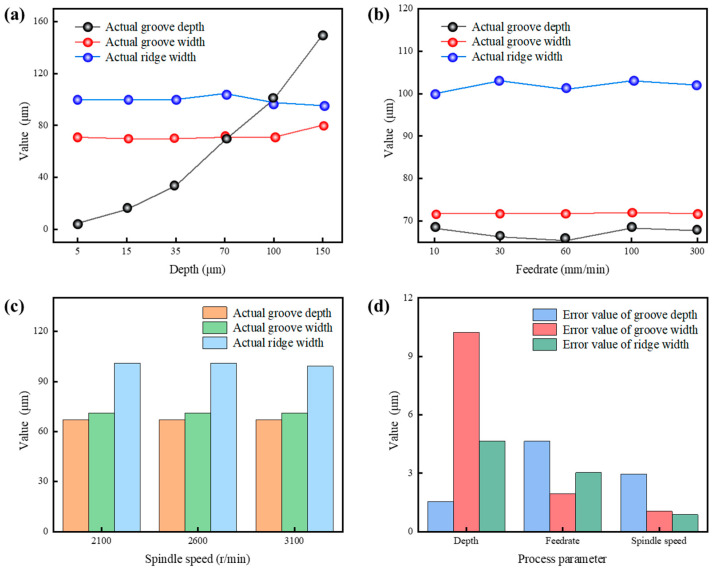
The effect of different process parameters on dimensional accuracy: (**a**) the effects of cutting depths on the actual groove depth, groove width, and rib width of rectangular grooves; (**b**) the effects of feedrate on the actual groove depth, groove width, and rib width of rectangular grooves; (**c**) the effects of spindle speed on the actual groove depth, groove width, and rib width of rectangular grooves; (**d**) the error values of the actual dimensions of rectangular grooves under different process parameters.

Figure 17 shows the relationship between the actual groove dimensions and the process parameters of the microstructure. As the cutting depth increases, the groove width and rib width of the rectangular groove are generally consistent with the theoretical values. Below a cutting depth of 70 μm, the actual cutting depth is slightly smaller than the theoretical depth. In their prior work, Cheng et al. [12] found that the actual depth was less than the theoretical depth during microstructure processing on the surfaces of cyclic olefin copolymers; the deviation between them was around 0.6 μm. This discovery is consistent with the results in this study. This may be due to the elastic recovery of the polymers, such as cyclic olefin copolymers and PI.

The variations in feedrate and spindle speed have a minimal impact on the rectangular dimensions. Figure 17d shows the maximum values of the dimensional errors for different parameters. It can be observed that the cutting depth has the greatest impact on the actual groove depth, with an error value of 10.23 μm. The next largest effects are those from the cutting depth on the rib width and the feed speed on the groove depth, both with an error value of 4.65 μm. This indicates that, among the process parameters, cutting depth has the most significant impact on dimensional accuracy, followed by feed speed, while spindle speed has the least effect on dimensional accuracy.

### 4.3. Roughness

**Figure 18:**
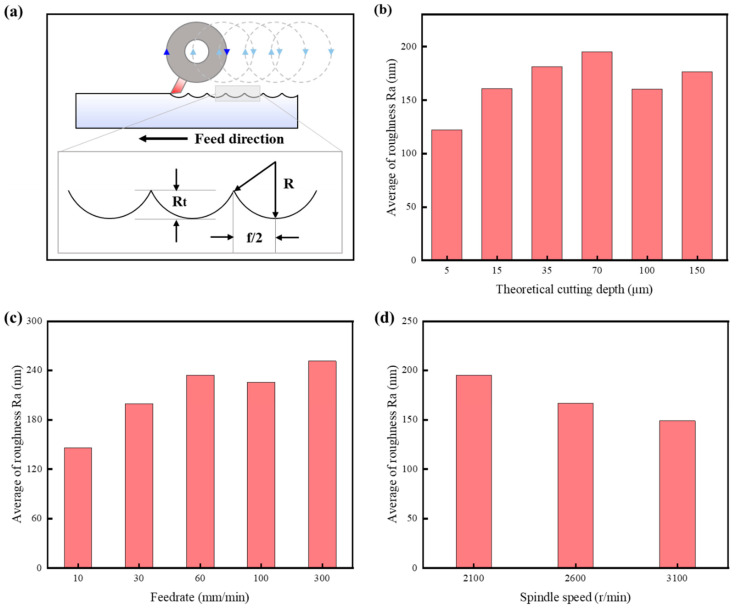
The effect of different process parameters on surface roughness: (**a**) formation mechanism of roughness; (**b**) cutting depth; (**c**) feedrate; (**d**) spindle speed.

Roughness is an important indicator of machining quality. According to the study by Cheng et al. [76], the roughness in UPFC is related to the cutting strategy, specifically the horizontal and vertical cutting approaches. Since the cutting strategy employed in this study is horizontal, only the roughness along the feed direction is analyzed. The maximum peak-to-valley height (R_t_) is a key parameter that affects surface roughness. Based on the cutting mechanism and strategy in UPFC, it is known that the maximum peak-to-valley height (R_t_) is related to the feed speed and the rotational radius (R), as shown in Figure 18a. Therefore, the maximum peak-to-valley height can be expressed as follows:

(15) $$R_{t}=R-\sqrt{R^{2}-{\left(\frac{f}{2}\right)}^{2}}$$

where R is the swing radius of the diamond tool and f is the step-over.

The figure clearly shows that surface roughness increases as the cutting depth increases, and that it gradually decreases as the spindle speed increases, which aligns with the theoretical calculations presented in Equation (3). However, in actual UPFC machining, surface roughness is influenced by multiple factors, including material properties, tool geometry, and process parameters such as cutting speed, feedrate, and environmental conditions. Among these factors, cutting depth plays a particularly critical role in determining surface roughness. A detailed analysis reveals that as the cutting depth increases, surface roughness exhibits a noticeable upward trend, indicating its substantial impact on surface quality. This phenomenon can be attributed to the fact that a greater cutting depth results in a higher material removal rate, leading to increased cutting forces. The higher cutting forces, in turn, may cause tool deflection, induce vibrations, and generate microstructural defects on the machined surface, thereby further deteriorating surface roughness [77,78].

### 4.4. Cutting Force

In the UPFC, the diamond tool continuously rotates at high speed, intermittently engaging and disengaging with the workpiece surface, resulting in discontinuous contact between the tool and the workpiece. In each rotational cutting cycle, the cutting duration is closely related to the rotational distance, cutting depth, and other cutting parameters. Analysis clearly shows that the first captured cutting force signal is a pulse, while subsequent vibration signals are induced by the cutting force, representing free vibrations, as the diamond tool has already left the workpiece surface.

**Figure 19:**
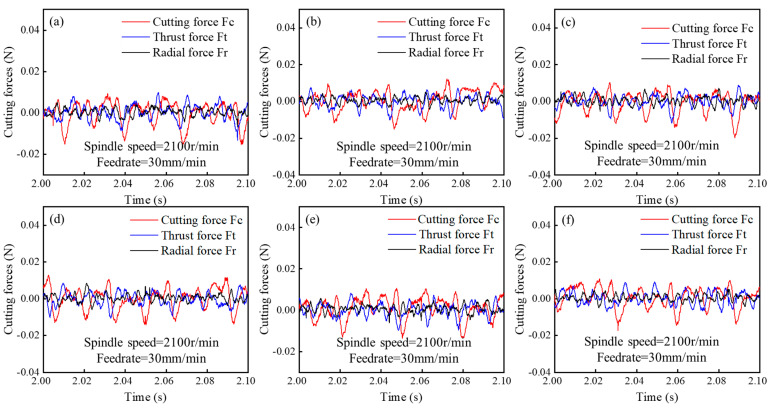
Cutting forces at different cutting depths: (**a**) 5 μm, (**b**) 15 μm, (**c**) 35 μm, (**d**) 70 μm, (**e**) 100 μm, (**f**) 150 μm.

**Figure 20:**
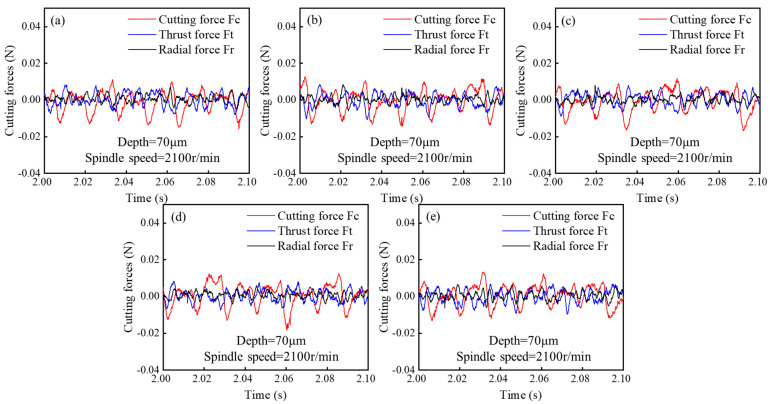
Cutting forces at different feedrates: (**a**) 10 mm/min, (**b**) 30 mm/min, (**c**) 60 mm/min, (**d**) 100 mm/min, (**e**) 300 mm/min.

**Figure 21:**
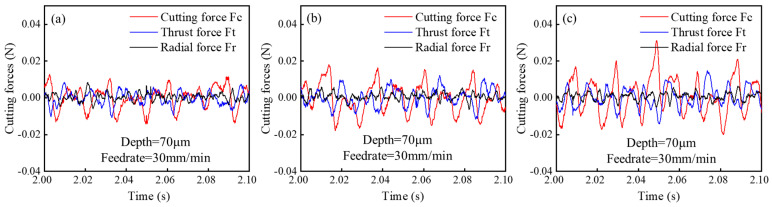
Cutting forces at different spindle speeds: (**a**) 2100 r/min, (**b**) 2600 r/min, (**c**) 3100 r/min.

As with other cutting processes, the selection of UPFC parameters directly affects the magnitude of the cutting forces. Figure 19 shows the time variation in the cutting force amplitudes in the feed, thrust, and radial directions at different cutting depths. The cutting force in the feed direction (F_c_) exhibits the largest amplitude, followed by the thrust force (F_t_), while the radial force (F_r_) shows the smallest amplitude. Figure 20 illustrates the variation in the amplitudes of the three cutting forces at different feedrates, with a trend similar to that of cutting depth. Figure 21 presents the amplitude variations in the three cutting forces at different spindle speeds.

**Figure 22:**
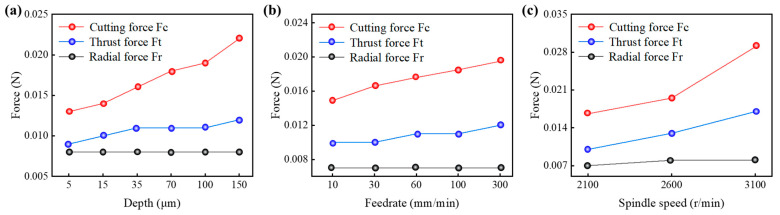
Curves of cutting forces at different process parameters: (**a**) cutting depths, (**b**) feedrate, (**c**) spindle speed.

The results show that as the cutting depth, feed speed, and spindle speed increase, the cutting forces in both the feed and thrust directions also increase. The radial force remains largely unchanged with variations in cutting depth and feed speed; however, it slightly increases with higher spindle speeds. This increase is attributed to the higher spindle speed, which amplifies the vibrations during the tool’s cutting process [79,80,81]. Figure 22 shows the variation in the trends of cutting forces with respect to the process parameters. It can be observed that the cutting force in the feed direction (F_c_) and the thrust force (F_t_) are the two key forces in fly-cutting, with the feed-direction force consistently being the largest, followed by the thrust force, and the radial force remaining the smallest.

Additionally, it is observed that the cutting force in the feed direction (F_c_) is particularly sensitive to variations in the process parameters, making it a key factor in assessing the machining dynamics of UPFC. Among the three cutting force components, Fc exhibits the most noticeable fluctuations in response to changes in cutting depth, feedrate, and spindle speed. Figure 22c presents the relationship between spindle speed and cutting force, providing further insight into this dependency. According to the theoretical analysis in Section 2.4, an increase in spindle speed is expected to reduce the cutting force. This is primarily because a higher spindle speed leads to a lower material removal rate per cutting pass, thereby decreasing the resistance encountered by the tool. As a result, the overall cutting force is reduced, contributing to an improved machining stability and potentially enhancing the surface quality. However, the experimental results do not align with this theoretical expectation. This discrepancy can be attributed to the fact that as the spindle speed increases, the tool’s linear velocity increases, resulting in a higher momentum. Consequently, the instantaneous impact force when the diamond tool contacts the workpiece also increases.

### 4.5. Chip Morphology

**Figure 23:**
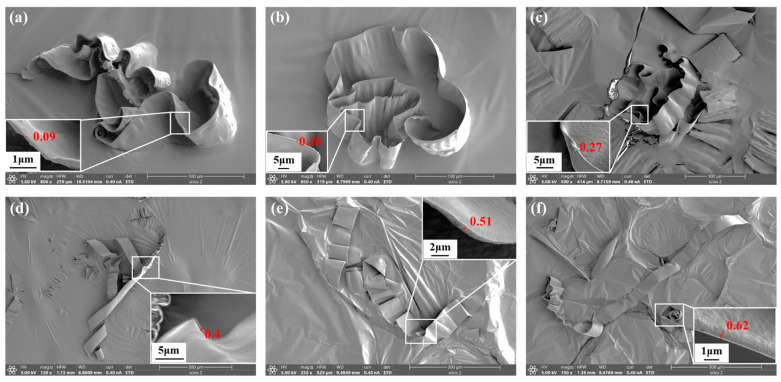
Chip thickness under different cutting depths: (**a**) 5 μm, (**b**) 15 μm, (**c**) 35 μm, (**d**) 70 μm, (**e**) 100 μm, (**f**) 150 μm.

**Figure 24:**
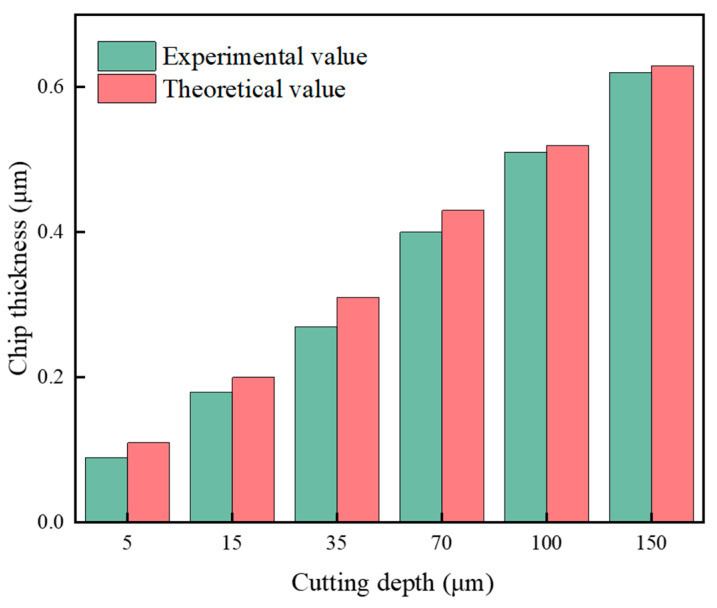
The experimental and theoretical values of chip thickness at different cutting depths of 5 μm, 15 μm, 35 μm, 70 μm, 100 μm and 150 μm, respectively. The feed speed is 30 mm/min, and the spindle speed is 2100 r/min.

Figure 23 presents the chips formed under different cutting depths in UPFC. It can be observed that the chips produced by UPFC are thin and long, with smooth, curved edges and no serrated features. Additionally, the chip morphology remains largely unchanged as the cutting depth increases. The observed chip morphology in UPFC indicates stable machining performance. According to the machining principle of UPFC, chips are removed smoothly during the process [48]. Due to the thin, curled, and overlapping characteristics of UPFC chips, accurately measuring their thickness is challenging. Therefore, for each cutting depth, a single measurable location is selected for evaluation, yielding chip thicknesses of 0.09 μm, 0.18 μm, 0.27 μm, 0.40 μm, 0.51 μm, and 0.62 μm, respectively. Figure 24 compares the measured chip thicknesses with the theoretical maximum chip thicknesses. The measured values are found to be smaller than the theoretical values, likely because the measurement positions do not correspond precisely to the maximum chip thickness. However, the overall trend of the measured and theoretical values is consistent as the cutting depth increases, demonstrating the accuracy of the maximum chip thickness model.

## 5. Conclusions

This study systematically investigated the ultra-precision fly-cutting of polyimide (PI) for microstructure fabrication, yielding several critical insights:

(1) Climb cutting outperformed conventional cutting in PI machining, producing a 20 nm lower surface roughness and significantly reducing burr formation at the groove edges, while also exhibiting lower cutting forces in the feed direction.

(2) Cutting depth emerged as the dominant parameter influencing dimensional accuracy: at the maximum depth of 150 μm, the groove width exceeded the theoretical value by 10.23 μm, while the rib width decreased by 4.65 μm.

(3) Cutting forces remained at the mN level but showed distinct trends: feed and thrust forces increased with cutting depth, feedrate, and spindle speed, whereas radial forces remained stable. Notably, when spindle speed exceeded 2600 r/min, feed-direction forces rose sharply due to increased impact effects.

(4) This study confirmed process stability across parameter variations, with feed speed and spindle speed showing negligible effects on dimensional accuracy and surface morphology. The established maximum chip thickness model, despite measurement challenges from chip curling, provided fundamental insights into PI’s material removal mechanism.

## Figures and Tables

**Table 1:** Mechanical and physical properties of PI.

**Parameters**	**Values**
Tensile strength (MPa)	85
Compressive strength (MPa)	160
Tensile modulus (GPa)	2.9
Compressive modulus (GPa)	1.5
Density ρ (kg/m3)	1430
Specific heat capacity (J/kg/°C)	1130
Thermal conductivity (W/m.°C)	0.35
Dielectric constant	3.32
Glass transition temperature (°C)	250

**Table 2:** Parameters of the machining system.

**Types**	**Contents**
X/Y/Z axis	The feedback resolution: 2.5 nm. The repeat positioning accuracy: X < 0.5 µm/100 mm, Y < 1 µm/100 mm, and Z < 0.2 µm/100 mm.
C axis	The feedback resolution: 2.5 nm. The rotation accuracy: <50 nm (radial) and 30 nm (axial).
Fly-cutting spindle	The rotation accuracy: <100 nm (radial). Radial gap stiffness at the front/middle/rear: 250/170/100 N/µm. Axial gap stiffness: 450 N/µm. Maximum speed: 20,000 r/min.

**Table 3:** Experimental process parameters.

**Types**	**Contents**
Machine tool	Width: 70 μm, rake angle: 0°, clearance angle: 3°
Cutting depth (μm)	5, 15, 35, 70, 100, 150
Feedrate (mm/min)	10, 30, 60, 100, 300
Fly-cutting spindle speed (r/min)	2100, 2600, 3100

**Table 4:** Performance parameters of Interferometer.

**Types**	**Contents**
Camera resolution (MP)	5
Camera scanning speed (kHz)	2
Objective/magnification (×)	5
Max. scanning range (μm)	400
Max. scanning speed (μm/s)	400
Topography reproducibility (nm)	<0.1

## Data Availability

The original contributions presented in this study are included in the article. Further inquiries can be directed to the corresponding author.
